# Operative Surgery, Adapted to the Living and the Dead Subject

**Published:** 1873-04

**Authors:** 


*Operative Surgery, adapted to the Living and the Dead subject.* By C. F. Maunder, Surgeon to London Hospital, etc. Second Edition, with one hundred and sixty-four illustrations. Philadelphia: Lindsay & Blakiston, 1873. Buffalo: T. Butler & Son.

It is now several years since Mr. Maunder's hand-book was first published. The first edition although not claiming any originality, was well received by the profession and especially so by students of operative surgery.

The second edition is somewhat increased in size, and has been improved in several respects. The author claims to have presented a new method of exsecting the elbow joint and of removing the lower jaw.

In exsections of the elbow, he makes a longitudinal incision on the back of the limb extending a short distance above and below the olecranon. After removal of the olecranon the joint is easily reached, examined, and its diseased parts removed. The point however upon which the author places particular stress is the preservation of the outer fibers of the triceps muscle, which by the attachment to the fore-arm, assist materially in making extension of the fore-arm.

The results claimed for this operation are very successful. As to the originality of this method of procedure if we mistake not, it was first advanced in 1864 by an American Surgeon, and has, to our knowledge been practiced in this country for some years.

The author's method of exsection of the lower jaw is to remove that bone through the mouth thus obviating the necessity to cutting the cheek and causing a deformity of the face. In certain cases this procedure seems feasible, but in many others it would be next to impossible. The author has produced a work which as a hand-book for those investigating operative surgery, is very valuable. To students in search of such a work we recommend this volume.

